# Patient counseling across neurologic subspecialties—a single academic center study

**DOI:** 10.3389/fneur.2026.1884883

**Published:** 2026-10-09

**Authors:** Raed Hailat, Benjamin Templeton, Nicholas Capaci, Andrew George, Ankit Annapareddy, Kevin Kerber, James Burke

**Affiliations:** Department of Neurology, The Ohio State University, Columbus, OH, United States

**Keywords:** counseling, education, neurology, outpatient care, subspecialty

## Abstract

**Background and objectives:**

Counseling is a routine part of neurologic care, but little is known about how commonly it is documented. We sought to characterize counseling in routine neurologic care by determining the proportion of visits with documented counseling, describe forms of counseling, and investigate factors that predict documentation of counseling.

**Materials and methods:**

We performed a cross-sectional study via retrospective chart reviews of 994 neurologic subspeciality outpatient encounters at a tertiary academic medical center from September 2023 to October 2023. The main outcome of this analysis was documented counseling (Yes/No) which was established via protocol-driven pre-specified chart review with assessment of abstraction reliability. Secondary outcomes included lifestyle modification (e.g., exercise, sleep, diet, alcohol, safety) and medication/disease specific education counseling (e.g., headache, seizure, medication, cognitive, stroke). We used a multivariable generalized estimating equations regression model with clustering at the provider level to compare how patient counseling varied across provider subspecialities.

**Results:**

Out of 994 outpatient encounters, 38% (*n* = 374) of visits had any documented counseling. Counseling on medication/disease specific education (28%, *n* = 276) was more common than on lifestyle education (6%, *n* = 60). The neurologic subspecialties with highest percentage of documented counseling were headache (73%), epilepsy (51%), and stroke (41%) while the neurologic subspecialties with the lowest percentage of any documented patient counseling were neuromuscular (19%), cognitive (15%), and multiple sclerosis (11%). Headache subspecialists were the only provider group to have increased odds of counseling (OR = 3.6, CI: 1.3–9.9) compared to general neurologists, while cognitive and multiple sclerosis subspecialists had 73% (CI: 42%−78%) and 72% (CI: 25%−89%) lower odds of documenting any counseling. Patient demographics were not statistically significantly associated with frequency of counseling.

**Discussion:**

Our study demonstrated significant variability in frequency and types of counseling documented across neurologic outpatient encounters and between subspecialists. The findings suggest that patient counseling may be undocumented and/or under-employed in certain fields. Future multi-centered studies are needed to understand documentation practices, actual performance of counseling relative to documentation, and patient clinical outcomes after participating in counseling.

## 1 Introduction

A neurological subspecialist visit can consist of a wide array of diagnostic, therapeutic and counseling activities, resulting in a variety of post-visit steps ranging from symptom management to diagnostics to definitive treatment (1). Diagnosis and treatment tend to be the focal points for training, research, and documentation. Counseling, on the other hand, can be a critical part of providing valuable clinical care (2). Counseling is the interaction between the provider and patient which aims to address overall health, education, and accommodations (2). It does this by promoting lifestyle modifications, expectations, occupational and family planning, and improved understanding of diagnosed conditions, prognoses, and treatment regiments (3–8). These components of patient care should be included in the electronic medical record as part of provider templates and/or personalized patient charts for the purposes of thorough documentation and billing.

However, there is insufficient evidence into the prevalence in which counseling is occurring for neurologic patients. For example, a 2022 retrospective cross-sectional analysis of the National Ambulatory Medical Care Survey (NAMCS) found diet counseling rates for post-stroke patients ranged from 5%-18% in the span of 2011–2016 with no clear trend over time (9). However, the NAMCS sample relied heavily on self-reporting by physicians which has been demonstrated to have low sensitivity for detecting health counseling behavior (10). Thus, its highly likely that prior samples utilizing tools like NAMCS are demonstrating underreporting of counseling for neurologic patients (10).

Given that counseling is intrinsic to patient-provider interactions and based on the lack of understanding of the role counseling serves in neurologic outpatient encounters, a sensible first step is to characterize the frequency and nature of counseling in routine care and between subspecialists. By sampling from a large tertiary academic medical center with a variety of neurologic conditions and neurologic subspecialities, our descriptive study sought to characterize the proportion of visits where education and counseling was documented in outpatient neurologic visits, what types of education/counseling were provided, and how both the proportion and type of education vary across subspeciality and credentials. The goal of this analysis was to begin the process of identifying opportunities to improve neurologic counseling to better inform providers in how counseling may be under-documented and/or underutilized in certain subspecialties to inform where future counseling and education interventions are most likely to be beneficial to improve value of care and patient outcomes.

## 2 Materials and methods

This research adheres to Strengthening the Reporting of Observational Studies in Epidemiology (STROBE) reporting guidelines. This cross-sectional study is based on data collected via retrospective chart reviews of 994 consecutive new outpatient encounters from neurologic providers at a large tertiary care academic medical center from September 2023 to October 2023. Data was abstracted via trained abstractors following a standardized abstraction protocol and after evaluating abstraction reliability across providers of all chart review elements. The abstractors were blinded to the subspecialties of the providers. For each encounter, the relevant neurology provider note was the input into the abstraction process which focused on the assessment and plan sections of each encounter. Additionally, other factors such as demographics and health provider information were abstracted from standardized elements in the electronic medical record.

### 2.1 Outcomes

The main outcome of this analysis was documented counseling (Yes/No) and secondary outcomes included lifestyle modification (i.e., exercise, orthostatic, sleep, diet, blood pressure, environment, alcohol, and safety) and medication/disease specific education counseling (i.e., headache, seizure, medication, cognitive, stroke, neuropathy, mental health, and other disease).

### 2.2 Chart review reliability

Chart reviews were conducted by three trained abstractors. Inter-rater reliability among the counseling variables was 0.99. Data on patient demographics and provider credentials were supplied by the honest broker.

### 2.3 Analysis

We reported on the descriptive statistics of the study sample's demographics including age, sex, race, and insurance coverage, in addition to information regarding provider subspecialty and their credentials. We examined patient counseling by reporting on the proportion of the study sample population that had any type of counseling, lifestyle modification, and medication/disease specific education counseling in general and stratified by provider subspeciality and credentials [MD; Doctor of Medicine, DO: Doctor of Osteopathic Medicine (a fully licensed physician just like an MD holder but with additional training in musculoskeletal system and whole person approach) (11), APP: advanced practice provider (a nurse practitioner or physician assistant who has completed graduate-level education) (12)]. To quantify the variation in primary and secondary outcomes explained by the provider and subspeciality levels we fitted ANOVA models and reported the R-squared. Primary and secondary outcomes were also analyzed using a multivariable generalized estimating equations regression models to compare how patient counseling varied across demographic variables and subspecialities and account for clustering at the provider level. Significance was determined at alpha = 0.05 level. Analysis was done using SAS 9.4 (Cary, NC).

### 2.4 Standard protocol approvals, registrations, and patient consents

This study was approved by the Institutional Review Board (IRB) at Ohio State University. This study used deidentified datasets; thus patient consent was not required.

### 2.5 Data access and availability

Deidentified data used in this research is not publicly available and is only accessible to the Ohio State University Medical Center faculty.

## 3 Results

### 3.1 Patient demographics

Across the 994 patients, the mean age was 52.9 (SD = 19) years, 36.9% (*n* = 367) were males, 79.7% (*n* = 792) participants identified as White (Table 1). The majority of the study sample were privately insured (45.3%, *n* = 450), 32.4% (*n* = 322) on Medicare, and 17.2% (*n* = 171) on Medicaid. The majority of patients (30.8%, *n* = 306) were seen by general neurologists and headache subspecialists (23%, *n* = 229). The lowest number of patients were seen by providers with a subspecialty in multiple sclerosis (5.4%, *n* = 54), cognitive (4.1%, *n* = 41), and neuropsychology (3.3%, *n* = 33). About 73% (*n* = 729) of the study sample were seen by providers with MD credentials.

**Table 1:** Descriptive statistics of the study population.

Variable		Number of patients (*N* = 994)	% or mean (SD)
Demographics			
Age		994	52.9 (19.0)
Sex	Male	367	36.9%
	Female	627	63.1%
Race	White (including Middle Eastern)	792	79.7%
	African American/black	118	11.9%
	African (other or Somali)	14	1.4%
	Asian (Chinese, Indian, Korean, Nepali, other)	24	2.4%
	Other	46	4.6%
Ethnicity	Non-Hispanic or Latino	941	94.7%
	Hispanic or Latino	35	3.5%
	NA	18	1.8%
Insurance	Managed care (private)	450	45.3%
	Medicare	322	32.4%
	Medicaid	171	17.2%
	Other Government	29	2.9%
	Self-pay	22	2.2%
Health provider credentials			
Neurology subspeciality visits/patients	Cognitive	41	4.1%
	Epilepsy	65	6.5%
	General neurology	306	30.8%
	Headache	229	23.0%
	Multiple sclerosis	54	5.4%
	Movement	69	6.9%
	Neuromuscular	89	9.0%
	Neuropsychology	33	3.3%
	Stroke	56	5.6%
	Other^*^	52	5.2%
Credentials	DO	196	19.7%
	MD	729	73.3%
	Advanced practice providers	69	6.9%

^*^Neurology resident, Neuro Otology, Neurogenetics, Neurophysiology, Pain, and Spinal.

### 3.2 Patient counseling subtypes

Of the 994 patient encounters, 37.6% (*n* = 374) of visits had documented counseling with 6.0% (*n* = 60) [16.0% of the visits with documented counseling] patients receiving documented lifestyle modification counseling and 27.8% (*n* = 276) [73.8% of the visits with documented counseling] receiving medication/disease specific education counseling. The most common form of lifestyle modification counseling was exercise (21.7%, *n* = 13) while the least common were diet and alcohol (6.7%, *n* = 4). The most common form of medication/disease specific education was headache (59.0%, *n* = 163), while the least common was mental health (1.8%, *n* = 5; Table 2).

**Table 2:** Granular types of patient counseling.

Variable	Counseling types	Number of patients (*N* = 994)	Proportion (%)
Any counseling	Yes	374	37.6%
	No	620	62.4%
Any lifestyle modification counseling	Yes	60	6.0%
	No	935	94.0%
Lifestyle modification counseling (*n* = 60)	Exercise	13	21.7%
	Orthostatic	10	16.7%
	Sleep	8	13.3%
	Diet	4	6.7%
	Blood pressure	6	10.0%
	Environment	9	15.0%
	Alcohol	4	6.7%
	Safety	6	10.0%
Any medication/disease specific education counseling	Yes	276	27.8%
	No	718	72.2%
Medication/disease specific education counseling (*n* = 276)	Headache	163	59.0%
	Seizure	44	15.9%
	Medication	13	4.7%
	Cognitive	9	3.2%
	Stroke	9	3.2%
	Neuropathy	13	4.7%
	Mental Health	5	1.8%
	Other disease	20	7.2%

### 3.3 Counseling stratification by provider subspecialty

The neurologic subspecialties with the highest percentage of patient visits with any documented patient counseling were headache (72.5%), followed by epilepsy (50.8%) and stroke (41.1%). The neurologic subspecialties with the lowest percentage of patient visits with any documented patient counseling were neuromuscular (19.1%), cognitive (14.6%), and multiple sclerosis (11.1%). The highest frequency of lifestyle modification counseling was observed in visits served by neuropsychology (18.2%) and stroke (12.5%) providers, while almost none was reported by multiple sclerosis or epilepsy providers. Medication/disease specific education counseling had the highest frequency among headache subspecialists (67.3%) and epilepsy subspecialists (43.1%) while the lowest frequency was seen in neuropsychology (6.1%) and multiple sclerosis (5.6%) subspecialties (Figure 1).

**Figure 1:**
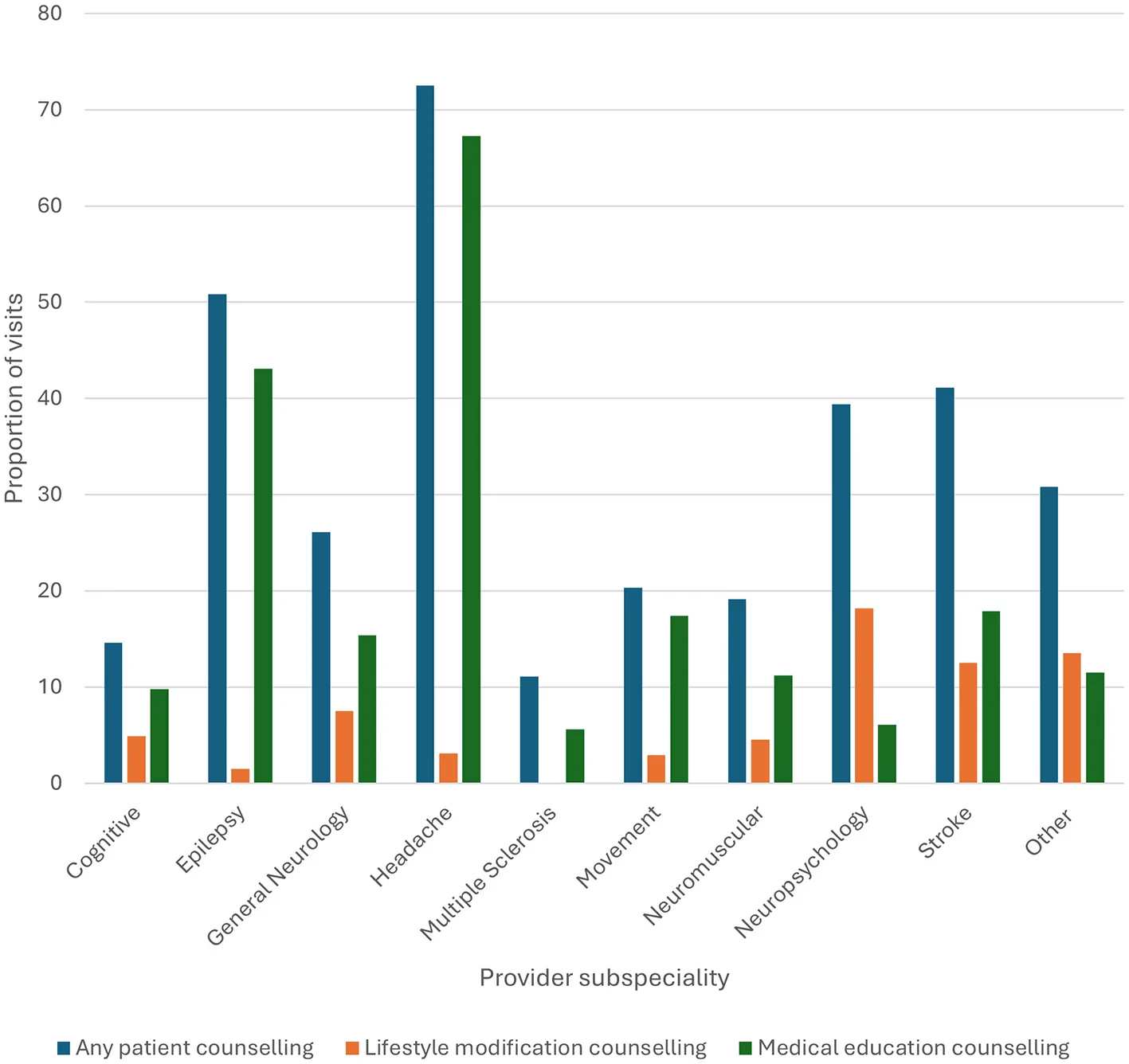
Patient counseling stratified by subspeciality and provider credentials. Other category includes Neurology resident, Neuro Otology, Neurogenetics, Neurophysiology, pain, and spinal cord.

Providers with MD credentials had the highest frequency of any documented patient counseling (42.9%) while those with DO credentials had the lowest (19.4%). The highest frequency of lifestyle modification counseling was observed in advanced practice providers (13.0%), followed by MD (5.8%). Medication/disease specific education saw the highest rates among MD-credentialed providers (32.8%) followed by advanced practice providers (18.8%).

A larger portion of the variance was explained by the providers rather than subspecialty. About 64% of medication/disease specific education counseling variance was explained by the provider credentials and subspecialty covariates (*R*-square = 64.3%). Provider credentials and subspecialty-level covariates explained a small amount of variance (*R*-square = 14.8%) in lifestyle modification counseling (Table 3).

**Table 3:** Proportion of variance explained by providers and subspecialties.

Outcome	Adjusted *R*-square		
	Provider level	Subspecialty level covariate	Provider and subspecialty level covariates
Any patient counseling	35.5%	19.0%	54.6%
Any lifestyle modification counseling	11.8%	3.1%	14.8%
Any medication/disease specific education counseling	38.0%	26.4%	64.3%

### 3.4 Adjusted analysis

Patient's demographics did not significantly change the odds of any counseling, lifestyle counseling or medication/disease specific education counseling (*p* > 0.05) (Table 4). Compared to general neurologists, increased odds of any type of counseling were only associated with visits seen by headache subspecialists (OR = 3.6, CI: 1.3–9.9), while patients seen by cognitive and multiple sclerosis subspecialists had 73% (CI: 42%−78%) and 72% (CI: 25%−89%) lower odds of receiving any patient counseling respectively. Compared to general neurology, no subspeciality was associated with increased odds of lifestyle modification counseling, but being seen by a headache subspecialist was associated with 71% (CI: 22%−89%) lower odds of lifestyle modification counseling compared to general neurology. Being seen by headache and epilepsy subspecialists were associated with 8.4 (CI: 3.4–20.9) and 3.2 (CI: 1.4–7.5) times higher odds of medication/disease specific education counseling respectively, however lower odds of medication/disease specific education counseling were associated with multiple sclerosis subspeciality (OR=0.30, CI: 0.09–0.99). Medical credentials were not associated with any increase in the odds of any counseling type, however patients seen by DOs had a 60% (CI: 5%-83%) lower odds of any type of counseling compared to patients seen by MDs (Table 4).

**Table 4:** Multivariate generalized estimated equations regression analysis of counseling.

Variable		Any patient counseling			Any lifestyle modification counseling			Any medication/disease specific education counseling		
		OR	95% CI	*p*-value	OR	95% CI	*p*-value	OR	95% CI	*p*-value
Patient demographics										
Age		0.99	0.98–1.01	0.49	1.00	0.98–1.02	0.82	0.99	0.98–1.01	0.20
Sex	Female	ref			ref			ref		
	Male	0.93	0.66–1.32	0.68	0.83	0.46–1.50	0.54	0.92	0.71–1.19	0.53
Race	White (including Middle Eastern)	ref			ref			ref		
	African American/black	0.77	0.49–1.23	0.27	0.90	0.36–2.22	0.81	0.78	0.47–1.29	0.33
	African (other or Somali)	0.66	0.26–1.65	0.38	1.51	0.17–13.54	0.71	0.68	0.32–1.43	0.31
	Asian (Chinese, Indian, Korean, Nepali, other)	1.07	0.54–2.14	0.85	1.34	0.29–6.30	0.71	1.12	0.77–1.62	0.56
	Other	1.12	0.63–2.00	0.71	1.67	0.52–5.38	0.39	1.20	0.56–2.56	0.64
Insurance	Managed care	ref			ref			ref		
	Medicaid	1.20	0.75–1.93	0.45	0.68	0.27–1.73	0.42	1.19	0.77–1.84	0.43
	Medicare	1.37	0.85–2.21	0.19	0.73	0.32–1.67	0.46	1.36	0.87–2.13	0.18
	Other Government	0.80	0.47–1.39	0.43	^**^	^**^	^**^	1.57	0.76–3.26	0.22
	Self-pay	0.90	0.31–2.59	0.84	1.01	0.20–5.19	0.99	0.93	0.29–3.06	0.91
Provider characteristics										
Subspeciality	General neurology	ref			ref			ref		
	Cognitive	0.27	0.13–0.58	0.00	0.52	0.10–2.56	0.42	0.53	0.20–1.44	0.21
	Epilepsy	1.81	0.72–4.55	0.20	0.16	0.02–1.31	0.09	3.24	1.40–7.50	0.01
	Headache	3.62	1.32–9.90	0.01	0.29	0.11–0.78	0.01	8.42	3.39–20.94	0.00
	Multiple Sclerosis	0.29	0.11–0.75	0.01	^**^	^**^	^**^	0.30	0.09–0.99	0.05
	Movement	0.44	0.17–1.20	0.11	0.31	0.06–1.52	0.15	1.07	0.47–2.47	0.87
	Neuromuscular	0.34	0.09–1.34	0.12	0.46	0.14–1.55	0.21	0.56	0.15–2.12	0.39
	Neuropsychology	1.34	0.22–8.09	0.75	2.00	0.65–6.15	0.23	0.35	0.06–2.08	0.25
	Stroke	1.33	0.36–4.95	0.67	1.48	0.52–4.209	0.47	1.08	0.45–2.58	0.87
	Other^*^	0.74	0.29–1.90	0.54	1.55	0.57–4.19	0.39	0.61	0.22–1.68	0.34
Provider credentials	MD	ref			ref			ref		
	DO	0.40	0.17–0.95	0.04	0.48	0.19–1.24	0.13	0.64	0.28–1.45	0.28
	Advanced practice providers	0.88	0.36–2.15	0.77	1.28	0.53–3.05	0.58	0.91	0.46–1.81	0.80

^*^Neurology resident, Neuro Otology, Neurogenetics, Neurophysiology, pain, and spinal cord.

^**^Categories not included in analysis because of zero cell count.

## 4 Discussion

This cross-sectional study based on data collected via retrospective chart reviews of outpatient encounters from neurologic providers at a large tertiary care academic medical center demonstrated significant variability in frequency and types of counseling utilized across neurologic visits and between subspecialists. The variation in the prevalence of documenting counseling across subspecialists is consistent with several possibilities: (1) difference in documentation between subspecialities, (2) different perceived utility of counseling generally, and (3) specific counseling types between subspecialities or provider-specific factors.

The different types of counseling utilized in our sample included lifestyle modifications (6.0%, *n* = 60) and medication/disease specific education counseling (27.8%, *n* = 276). Even though educating patients on medication use, adherence, and possible adverse events constitutes a major proportion of counseling, but we unexpectedly observed a low prevalence of prescription medication education (13). This may reflect undocumented counseling in our sample and raises the possibility that our sample underestimates true counseling rates, considering past studies have found counseling to occur in about 50% of outpatient primary care encounters although is also consistent with the possibility that counseling is truly performed less often in neurology visits (14). Meanwhile, the data collected regarding lifestyle counseling also indicates possible discrepancies about what may be documented vs. what may be left out of the electronic medical record. Of all subspecialties studied in this sample, only headache visits had a statistically significant (*p* = 0.014) increased likelihood of engaging in lifestyle modifications compared to general neurologists. This may reflect cultural norms and guidelines in headache care ranging from identifying specific headache triggers to improving hygiene, adequate hydration, and stress management (15, 16). However, it would also be expected for other specific subspecialties to engage in lifestyle modifications at a higher probability than general neurology outpatient encounters. For example, stroke is caused by vascular risk factors, diet, smoking and reduced physical activity, all of which may be amenable to counseling centered around lifestyle changes to prevent further brain injuries in the future (17). In fact, current American Heart Association/ American Stroke Association guidelines recommend educating stroke patients about behavioral modifications that can assist in cardiovascular risk factor reductions (18). Additionally, patients with movement and neuromuscular conditions may likely have significant modifications to their abilities to complete activities of daily living with necessary coaching from their providers to enhance patient safety (19). However, none of these specialties had documented education regarding lifestyles that were statistically different from general neurologists (stroke *p* = 0.466, neuromuscular *p* = 0.211, movement *p* = 0.149). This again points to possible disparities in charting patient counseling between subspecialists.

When considering differences in our sample of subspecialties, general neurologists and headache specialists had the highest frequency of visits in our sample, while cognitive and multiple sclerosis providers were among those with the fewest documented patient visits. Multiple explanations could be behind these differences, such as sampling bias, commonality of headache chief complaints in the larger population, as well as the need for more counseling regarding the risks, benefits, and outcomes of procedures in certain specialties (20). This variation could also be explained by the provider practice patterns which we have found to explain a higher proportion of the variance compared to the subspecialty alone. However, subspecialty stratification and the multivariable analysis that eliminated provider level practice patterns through clustering demonstrated the highest percentage of patient education counseling was among headache subspeciality and the lowest within multiple sclerosis and cognitive subspecialities. These subspeciality specific and not provider driven observations may be explained by the lower expectation or altered cognitive status of the patients which may de-incentivize documentation, the utilization of templates that does not include documentation, or simply a subspecialty cultural norm of under-utilization or under-documentation (21). It's also possible that reimbursement incentives may discourage providers prioritizing the documentation of counseling in the electronic health record (22). Additionally, as visit complexity and patient volume loads grow, neurologists may face limits in time to engage in educational discussions (23). Finally, certain conditions may be viewed as better managed with chronic pharmacological treatment methods and may not warrant continued patient education (24). Variation between subspecialties demonstrates need for further investigation of clinical outcomes after utilization of patient counseling. In terms of physician credentials, providers with MD credentials were most commonly sampled, followed by DO and advanced practice providers. Provider education background differences were evident as physicians with MD-credentials documented counseling most often, while DOs reported significant lower rates. Interestingly, advanced practice providers had the highest observed frequency of documented lifestyle modification counseling. This variation can be due to possible differences in training emphasis including graduate and residency institutions or practices and documentation behaviors.

### 4.1 Limitations

This study has several limitations. Due to the single center nature of the study, findings may not necessarily be generalizable to other clinical settings. Additionally, while this study overall had a large sample size, several neurology subspecialties had smaller representation, limiting the precision of these results. Furthermore, study results exclusively utilized documented counseling within the electronic medical record, which may underestimate the true frequency of education due to potential incomplete documentation. On the other hand, overestimation of counseling could have been the case in some subspecialities due to use of templates for documentation with less certainty about actual performance of the counseling (25, 26), but we were unable to ascertain this in our data or conduct provider surveys to assess their practice patterns. While inter-rater reliability was assessed at the onset of this study, variability in abstraction and interpretation of charts could possibly skew results. Finally, the cited references are quite dated, but it is a reflection of the literature itself hence this study provides a relative up to date analysis of counseling documentation.

This study highlights the documented patient counseling across neurologic subspecialties and credential types in a single tertiary academic medical center. The findings in this study suggest possible under-utilization and/or un-documentation of patient education in certain fields of neurology, while highlighting the emphasis on this component of patient care in others. The fairly low observed rates of documented counseling in many subspecialities also raises the possibility that counseling is not taking place during clinical encounters, a possibility that should be explored in future work. We believe that while these results may not answer a central question, they do inform subsequent research into counseling including the true rates of counseling and whether they are as low as documented, the quality of counseling, and how ought documentation of counseling most effectively be performed. Given the potential impact on patient outcomes, further investigation is needed to understand these questions and how providers choose to document counseling and how day-to-day barriers and facilitators may affect certain physicians' ability to engage in education. We recommend in the next steps of research the implementation of direct observational study design and mixed methods analytics to collect high-quality data during the medical encounter and include qualitative data from the providers. Additionally, multicenter studies incorporating provider surveys in addition to chart abstraction could help with generalizing these findings to a larger population.

## Data Availability

Deidentified data used in this research is not publicly available and is only accessible to the Ohio State University Medical Center faculty. Requests to access the datasets should be directed to raed.hailat@osumc.edu.
